# Gastric Perforation During MRI After Ingestion of Ferromagnetic Foreign Bodies

**DOI:** 10.5811/cpcem.2021.4.52307

**Published:** 2021-07-27

**Authors:** Nicholas M. Glover, Ryan Roten

**Affiliations:** Desert Regional Medical Center, Department of Emergency Medicine, Palm Springs, California

**Keywords:** Metallic foreign body, magnetic resonance imaging, gastric perforation

## Abstract

**Case Presentation:**

A 65-year-old male with schizophrenia and intellectual disability ingested what was reported to be two AA batteries, prior to a scheduled magnetic resonance imaging (MRI) study. He developed severe abdominal pain and presented to the emergency department the following day with hypovolemic/septic shock. General surgery retrieved two metal sockets and a clevis pin from the stomach prior to surgical repair of a gastric perforation. This case highlights a rare yet critical outcome of ingesting ferromagnetic foreign bodies prior to an MRI study.

**Discussion:**

Medical literature on this subject is scarce as indwelling metal foreign bodies are a contraindication to obtaining an MRI. Yet some patients with indwelling metallic foreign bodies proceed with MRI studies due to either challenges in communication such as age, psychiatric/mental debility, or unknowingly having an indwelling metal foreign body. In this case, the patient surreptitiously ingested metal objects prior to obtaining an MRI.

## CASE PRESENTATION

A 65-year-old, Spanish-speaking male with a history of schizophrenia presented to the emergency department hypotensive and diaphoretic complaining of severe abdominal pain. The patient was an exquisitely poor historian; however, we were able to ascertain that he recently had a routine outpatient magnetic resonance imaging (MRI) performed the day before, which was apparently halted due to the patient complaining of severe abdominal pain, and he was subsequently sent home. On further questioning, the patient admitted to ingesting two AA batteries prior to the MRI study because he “thought it would make him smarter.”

**Image 1:**
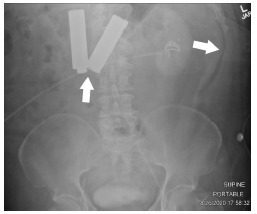
Plain film of the abdomen upon initial evaluation in the emergency department to indicate position of foreign bodies, approximately one day after the patient received magnetic resonance imaging (MRI). Left arrow indicates foreign bodies within the stomach, which were reported to be two AA batteries per the patient, ingested prior to the MRI study. Right arrow indicates free air within the abdomen suggesting hollow viscus perforation.

Initial workup included plain films of the abdomen, which demonstrated two radiopaque foreign bodies in the stomach possibly resembling AA batteries, per reported patient history, with associated pneumoperitoneum (Image 1).

**Image 2:**
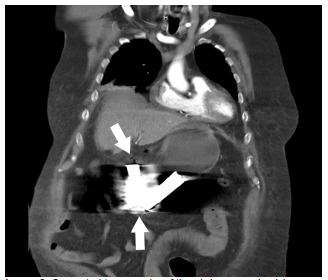
Computed tomography of the abdomen and pelvis demonstrating foreign bodies that appear to be in the stomach with extensive artifact due to metallic foreign bodies. Bottom arrow points to two foreign bodies, which were reported to be two AA batteries per the patient. Top arrow points to free air within the peritoneum suggesting hollow viscus perforation.

After resuscitation and general surgery consultation, computed tomography of the abdomen and pelvis was performed, which demonstrated presumed perforated hollow viscus injury with unclear perforation site due to extensive metallic artifact and pneumoperitoneum (Image 2).

**Image 3:**
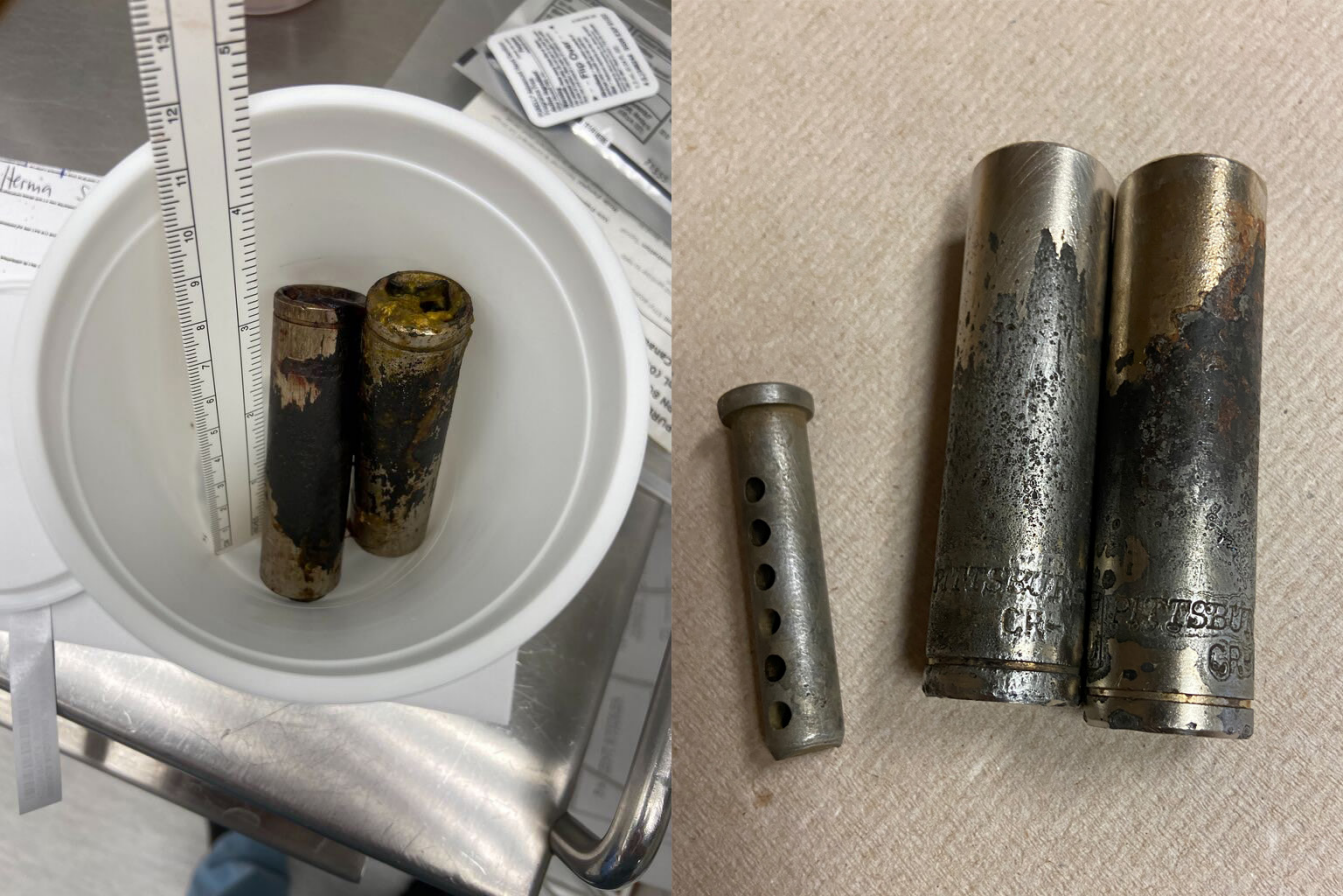
Images of the retrieved foreign bodies in the operating room, which were reported to be two AA batteries per the patient. Closer evaluation reveals foreign bodies are two, approximately 3.5-cm metal sockets typically used with a socket wrench and a clevis pin, which was nested within one of the sockets creating the illusion of an AA battery silhouette on plain film.

The patient was immediately taken to surgery for exploratory laparotomy where the foreign bodies were removed, and a three-centimeter (cm) defect in the body of the distal stomach and a small serosal stomach defect were repaired. The foreign bodies were then more clearly identified as two approximately 3.5-cm long metal sockets as well as a clevis pin, which was nested within one of the sockets (Image 3).

## DISCUSSION

Ferromagnetic and conductive metal fragments are subject to translational attraction and torque when under strong magnetic forces, which may ultimately lead to dislodgement or excessive heating.^1^ One case report discusses a three-year-old child obtaining an MRI of the head prior to sinus surgery where there was found to be extinction of the face on the first MRI image, but not appreciated on initial scout imaging.^2^ A button battery lodged within the nostril was identified on further physical inspection and removed. Another case report discusses a 65-year-old metal grinder complaining of acute severe left eye pain during MRI of the brain, which was immediately terminated.^3^ The patient unknowingly had a metal fragment in his eye, which was ultimately removed by an ophthalmologist. In these cases, MRI screening questioning failed due to the inability to obtain history from a child and unknowingly having an indwelling metal foreign body lodged in an eye, respectively.

**CPC-EM Capsule**

What do we already know about this clinical entity?

*It is well known that indwelling ferromagnetic materials are a contraindication to magnetic resonance imaging and reported complications due to this are rare*.

What is the major impact of the image(s)?

*The images in this case demonstrate the dangers of indwelling ferromagnetic materials while undergoing magnetic resonance imaging scan*.

How might this improve emergency medicine practice?

*This case suggests obtaining screening plain films in unreliable historians complaining of pain after magnetic resonance imaging*.

In the presented case, the patient surreptitiously ingested metal foreign bodies prior to obtaining a scheduled MRI of another body part, supposedly of the head. It is unclear why the patient was sent home after complaining of severe abdominal pain after an MRI, yet a scout image for the MRI head study likely would not have revealed the metal foreign bodies within the stomach. In such situations with unreliable historians, full body plain films may be beneficial to evaluate for foreign bodies as well as prompt ED referral in instances of pain during an MRI. Regardless, the patient presented in extremis, the diagnosis was promptly established, definitively corrected surgically, and he was discharged from the hospital tolerating oral intake two weeks later.
